# BONE HEALTH AFTER LUNG TRANSPLANTATION: A CASE REPORT

**DOI:** 10.2340/jrm.v58.46110

**Published:** 2026-07-23

**Authors:** Vanessa LEE, Paula BERMEL, James FOSTER

**Affiliations:** 1 School of Graduate and Professional Studies, Messiah University, Mechanicsburg, PA; 2 Department of Health and Rehabilitation Sciences, Temple University, Philadelphia, PA, USA

Among many possible adverse events after lung transplantation, osteoporosis and fragility fractures are common, serious complications. Low bone mineral density (BMD) is present in 84–86% of lung transplant recipients, with 25–37% qualifying as osteoporotic (1, 2). Loss of BMD is most pronounced in the first 3–6 months post-transplant (3). In the first 3 years following lung transplantation, 18–37% of recipients experience a fragility fracture, most commonly in the spine and ribs (2, 3). Although fragility fractures are more common in patients with low BMD, they were also present in 15% of lung transplant recipients with normal BMD, highlighting the need to consider factors beyond BMD (2, 4). Patients who experience a fragility fracture after lung transplantation have an increased risk of early mortality and a 4–7x increased risk of an additional fracture in the next year (4, 5). The purpose of this case report is to describe a patient who experienced multiple vertebral compression fractures (VCF) in the acute recovery period after bilateral lung transplantation and to use this case to inform how rehabilitation professionals may proactively incorporate bone health considerations into their patient management.

## CASE DESCRIPTION

A 69-year-old female was in the process of completing pre-transplant testing when she developed acute on chronic respiratory failure due to chronic obstructive pulmonary disease, requiring ventilator and extracorporeal membrane oxygenator support. Her past medical history was significant for chronic obstructive pulmonary disease, on 2 litres of oxygen at rest with intermittent BiPAP support, atrial fibrillation, osteoporosis, and secondary adrenal insufficiency. She was admitted to an academic medical centre where she completed the lung transplant workup and received a double lung transplant on the 69th day of her hospital stay. Twenty-eight days later, the patient was discharged to an acute rehabilitation centre, where she participated in physical and occupational therapy for almost 8 weeks before being readmitted to the hospital with severe back pain and shortness of breath. Medical imaging revealed bilateral multiloculated pleural effusions, which were managed with 2 pigtail chest tubes, and recent mild multilevel VCF (T5, L2, L5) in addition to chronic VCF at T3, T4, T6, and T8. The patient later recalled bending forwards to tie her shoes as the activity that precipitated her acute back pain. Her VCF were evaluated by the Orthopedic Surgery team, who recommended spinal precautions and a thoracic-lumbar-sacral orthosis when out of bed. On the 11th day of her readmission, the patient underwent vertebroplasty at L2 and L5 due to persistent pain. Four days later, the patient returned to the acute rehabilitation centre.

This patient was highly motivated and had excellent family support throughout her episode of care. She received physical and occupational therapy throughout her hospital stays, although the plan of care varied significantly based on her medical status and activity tolerance. She participated consistently in her therapeutic programme with a refusal rate of 4% (3 out of 80 attempted sessions). At the time of her final discharge to the acute rehabilitation centre, she was walking 15.3 metres (50 feet) x 4 with a rollator, minimal assistance, and seated breaks between each ambulation trial and the next one. Table I offers more details regarding the patient’s medical and rehabilitation episode of care.

**Table I:** Timeline of major medical and rehabilitation events

24–27 days prior to hospital admission	Outpatient Pretransplant Screen - Six Minute Walk Test distance = 120 meters (393.3 feet) with a dyspnoea rating of 8/10 (very severe) and leg fatigue of 8/10 (very severe) and dizziness - Short Performance Physical Battery 5/12 ○ Balance = 4 ○ Gait speed = 1 ○ Sit to stand = 0 (used arms) - Grip Strength ○ Right 13.3 kg ○ Left 12.7 kg - CT Thorax: Mild chronic compression fracture deformities at T3, T4, and anterior wedging of T8
Hospital day 1	Admitted with acute on chronic hypercapnic respiratory failure, requiring ventilator and extracorporeal membrane oxygenator support
Hospital day 4	Initial PT evaluation • Minimal assist to transfer to edge of bed, stand, and walk 100 ft (30.5 m) • Session limited by tachycardia and dyspnoea on exertion
Hospital day 6	Endocrine consult completed • Recommended DEXA scan when patient discharged • Acknowledging high risk for repeat vertebral compression fracture given high chronic steroid use • Due to prolonged hospital stay with multiple complications, DEXA scan was not completed until 3 months after transplant. Results are included below
Hospital day 6 – 28	7 PT sessions over 22 days, focused on transfer and gait training with more than half of sessions including active range of motion exercise
Hospital day 26	Initial OT evaluation completed; included both functional mobility and self-care assessments
Hospital day 29 – 33	PT and OT on hold due to medical instability, patient intubated and sedated
Hospital day 34 – 44	4 PT sessions and 1 OT session focusing on bed mobility and transfer to chair, self-care activities; sessions limited by tenuous medical status and poor activity tolerance
Hospital day 45	Percutaneous tracheostomy, had 1 PT session focusing on bed mobility
Hospital day 46 – 68	15 PT and 4 OT sessions over 21 days, focused on transfer training and self-care activities; very minimal tolerance for ambulation
Hospital day 69/Post transplant day (post-transplant day) 0	Double lung transplant, Simulect induction
Post-transplant day 2 – 6	PT and OT complete re-evaluations • In total, 4 PT and 3 OT sessions occur during this time • Sessions focus on bed mobility, transfer training, seated exercise, and self-care assessment and activities
Post-transplant day 7 – 28	19 PT and 2 OT sessions • PT sessions focus almost exclusively on gait training with seated breaks • OT sessions focus on self-care assessment and activities; limited by mild shortness of breath
Post-transplant day 25	Decannulated
Post-transplant day 28	Discharged to acute rehabilitation centre for 54 days
Post-transplant day 82	Readmitted to acute care hospital with back and chest pain, shortness of breath
Post-transplant day 83	2 chest tubes placed for transudative loculated pleural effusions Orthopaedic surgery consult completed and recommended TLSO brace for out of bed, weightbearing as tolerated bilateral upper and lower extremities, spinal precautions, and surgical intervention if severe pain persists
Post-transplant day 87	PT and OT evaluations completed with TLSO and spinal precautions, 2 chest tubes • 8/10 at mid to low back on visual analogue scale, “all the time” • Bilateral lower extremity strength: hip flexion: at least 3/5 (no resistance), knee extension: 4/5, dorsiflexion: 5/5 • Minimal assist with rollator to walk 7.6 m (25 ft) x 2 • Self-care with assistive devices; minimum assist for upper body including TLSO and maximum assist for lower body
Post-transplant day 88 – 95	5 PT and 3 OT sessions over 8 days
Post-transplant day 92	Vertebroplasty completed at L2 and L5 levels
Post-transplant day 96	Patient discharged back to acute rehab • Back pain at final PT session was 4/10 on the visual analogue scale • Walking 15.2 m (50 ft) x 4 (seated breaks) with rollator, minimal assist • Self-care with assistive devices.
Post-transplant day 99	DEXA bone scan completed at L1 – L4 (excluding L2 due to cement) • Left hip = total bone mineral density of 0.502 g/cm^2^ corresponding to T-score of –3.6 • Left femoral neck = bone mineral density of 0.44 g/cm^2^ corresponding to a T-score of –3.3 • Total lumbar spine (excluding L2) demonstrated a total BMD of 0.655 g/cm^2^ corresponding to a T-score of –3.6

CT: computed tomography PT: physical therapy; DEXA: dual-energy X-ray absorptiometry; OT: occupational therapy; m: meter; ft: feet; TLSO: thoracic-lumbar-sacral orthosis.

## DISCUSSION

This case report describes a lung transplant recipient who experienced multilevel VCF during acute rehabilitation, which necessitated hospital readmission and eventual vertebroplasty after conservative management did not result in adequate symptom relief. Although the risk factors were obvious in retrospect, the VCF was unexpected at the time it occurred.

### Risk factors

Many published risk factors for VCF after lung transplant were present for the patient in this case. Please refer to Table II for details. Starting 2 years prior to her admission date, this patient continuously required glucocorticoid medication for pulmonary disease, which is well known to increase bone resorption and diminish bone formation and remodelling (3). Additionally, her pre-transplant score on the Short Performance Physical Battery (SPPB) of 5/12 was consistent with frailty. (2) Lung transplant recipients scoring ≤ 9 on SPPB have a 2-fold increased risk of fracture, and for each point lower, the fracture risk increases by 15% (2).

**Table II:** Risk factors for vertebral compression fracture in lung transplant recipients

Risk categories	Risk factors
Lung transplant specific factors (3)	Diagnosis of COPD* or cystic fibrosis Chronic hypoxia and/or hypercapnia* Glucocorticoids* Calcineurin inhibitors (cyclosporine or tacrolimus)* Immobilization*
Personal factors (2, 3)	Higher age* Frailty* Vitamin D deficiency Diabetes mellitus Pre-existing bone disease* or fracture* Malnutrition Lower BMI Postmenopausal status* Genetic risk
Lifestyle factors (3)	Tobacco use Alcohol consumption Sedentary lifestyle*

* Indicates this risk factor was known to be present for the patient described in this case report at the time of her hospital admission.

COPD: chronic obstructive pulmonary disease; BMI: body mass index.

### Suggested modifications for rehabilitation protocols

**Fig. 1:**
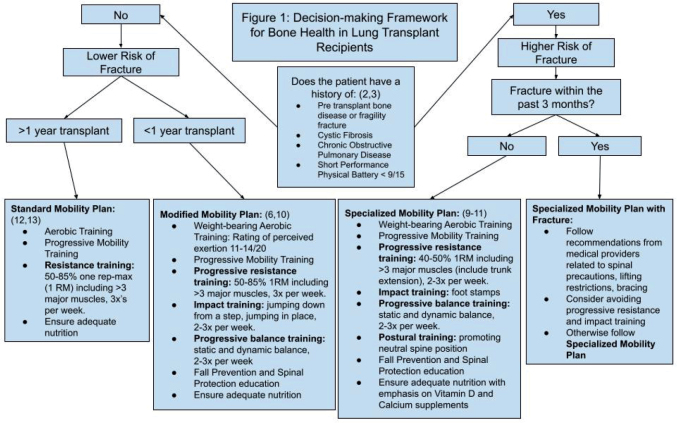
Decision-making framework for bone health in lung transplant recipients.

Tailoring rehabilitation protocols to include consideration for bone health and mitigation for fracture risk should be a priority for all rehabilitation professionals working with lung transplant recipients, especially during the first year post-transplant when loss of BMD and fracture risk are the highest (3, 4). Fig. 1 presents an algorithm to support clinical decision-making by fracture risk level. This algorithm contains evidence-based recommendations that may be adapted according to facility, programme, or department needs.

### Educational components

Spinal protection education teaches the patient to limit the biomechanical load placed on vertebral bodies by limiting trunk flexion, especially combined with rotation or completed in a rapid or repetitive manner. Patients should avoid lifting that requires full body strength or lifting from a position of spinal flexion (6). Postural education is also important as bone is susceptible to injury from chronic static load and creep even in the absence of an acute inciting injury (7).

Excessive spinal flexion during self-care activities should be avoided (6, 8). Occupational therapists can recommend activity modification using long-handled assistive devices for dressing, bathing, and item retrieval, to reduce spinal loading while maintaining independence in self-care activities.

Fall prevention education is essential for avoiding fall-related injuries and is well described in rehabilitation literature (2).

### Therapeutic exercise

For physical training to support bone health, the mechanical load must be adequate to stimulate bone formation and remodelling; loading should start low with feedback for proper technique and be increased gradually (6, 9). Loading patterns should favour dynamic activities over static and include diverse loading patterns and adequate rest (9). As with all training programmes, targeted areas will be the most directly affected, cessation of routine loading will result in reversal of the benefits, and those with the lowest initial BMD values have the greatest potential for increased bone formation (9).

The exercises in Fig. 1 highlight the combination of resistance training and impact exercise, which is most effective for improving bone health (6, 10). Muscle strengthening and joint conditioning should be completed prior to or concurrent with impact exercise to mitigate injury risk (6). Impact training should start at low intensity and be gradually progressed; high-intensity impact training will not be appropriate for all patients (6). Balance training should include dynamic and static exercises and become more challenging as tolerated with the goal of high-intensity training (6, 9). Back extension exercises can improve spinal posture and mobility as well as the strength, recruitment, and activation of erector spinae muscles (6, 11).

This case report exemplifies the risk for VCF after lung transplant that is well described in the medical literature but almost absent from rehabilitation literature. Limitations include the retrospective nature of the data collection and the varied treatment priorities of the 15 physical therapy professionals and 5 occupational therapy professionals who participated in this patient’s care. All case reports have limited generalizability of specific case information.

In conclusion, rehabilitation protocols for lung transplant recipients should address bone health. Lung transplant recipients are at increased risk of fragility fracture, even in the setting of normal bone mineral density. Mitigation of the risk for vertebral compression fractures should include both education and individualized training. More research is needed to determine the feasibility and effectiveness of these recommendations.
